# Safety, Protective Immunity, and DIVA Capability of a Rough Mutant *Salmonella* Pullorum Vaccine Candidate in Broilers

**DOI:** 10.3389/fmicb.2017.00547

**Published:** 2017-04-05

**Authors:** Rongxian Guo, Yang Jiao, Zhuoyang Li, Shanshan Zhu, Xiao Fei, Shizhong Geng, Zhiming Pan, Xiang Chen, Qiuchun Li, Xinan Jiao

**Affiliations:** Jiangsu Key Laboratory of Zoonosis, Jiangsu Co-Innovation Center for Prevention and Control of Important Animal Infectious Diseases and Zoonoses, Yangzhou UniversityYangzhou, China

**Keywords:** *Salmonella* Pullorum, live attenuated vaccine, DIVA, lipopolysaccharides, cross-protection

## Abstract

*Salmonella enterica* subsp. *enterica* serovar Gallinarum biovar Pullorum (*Salmonella* Pullorum) is highly adapted to chickens causing an acute systemic disease that results in high mortality. Vaccination represents one approach for promoting animal health, food safety and reducing environmental persistence in *Salmonella* control. An important consideration is that *Salmonella* vaccination in poultry should not interfere with the salmonellosis monitoring program. This is the basis of the DIVA (Differentiation of Infected and Vaccinated Animals) program. In order to achieve this goal, *waaL* mutant was developed on a *spiC* mutant that was developed previously. The safety, efficacy, and DIVA features of this vaccine candidate (*Salmonella* Pullorum Δ*spiC*Δ*waaL*) were evaluated in broilers. Our results show that the truncated LPS in the vaccine strain has a differentiating use as both a bacteriological marker (rough phenotype) and also as a serological marker facilitating the differentiation between infected and vaccinated chickens. The rough mutant showed adequate safety being avirulent in the host chicks and showed increased sensitivity to environmental stresses. Single intramuscular immunization of day-old broiler chicks with the mutant confers ideal protection against lethal wild type challenge by significantly stimulating both humoral and cellular immune responses as well as reducing the colonization of the challenge strain. Significantly lower mean pathology scores were observed in the vaccination group compared to the control group. Additionally, the mutant strain generated cross-protection against challenge with the wild type *Salmonella* Gallinarum thereby improving survival and with the wild type *Salmonella* Enteritidis thereby reducing colonization. These results suggest that the double-mutant strain may be a safe, effective, and cross-protective vaccine against *Salmonella* infection in chicks while conforming to the requirements of the DIVA program.

## Introduction

In poultry, the infectious bacteria *Salmonella* *enterica* subsp. *enterica* can be divided in two broad groups of serovars on the basis of pathogenesis and infection biology (EFSA, 2004). One group, containing the serovar Gallinarum biovars Pullorum and Gallinarum, causes a severe systemic typhoid-like disease in a restricted range of hosts (Barrow and Freitas Neto, 2011). The other group, containing the serovar Enteritidis, causes gastrointestinal disease in a wide range of hosts including humans. *Salmonella* Pullorum is highly adapted to young chicks under 3 weeks of age, and results in acute systemic disease and high mortality. In many developing countries, *Salmonella* Pullorum infections in poultry are common and pullorum disease remains the principal disease threat to the fowl industry (Guo et al., 2016). *Salmonella* Enteritidis is the cause of the food-borne salmonellosis pandemic in humans, in part because it has the unique ability to contaminate poultry products without causing discernible illness in the birds infected (Guard-Petter, 2001). Therefore, there is a need for methods that protect broilers, from day-of-hatch until slaughter age, against infection with *Salmonella* Pullorum, as well as to reduce the contamination of the food-borne serotype *Salmonella* Enteritidis.

Vaccination of chicks has offered a promising future and there continues to be progress in the development of a safe and efficacious *Salmonella* vaccine that provides broad cross-protection for enhancing both animal health and food safety (Heithoff et al., 2015). *Salmonella* Pullorum is likely to be eliminated from poultry solely by relying on the test-and-slaughter method of disease control (World Organization for Animal Health [OIE], 2008). Pullorosis sero-diagnosis is generally based on the detection of antibodies against *Salmonella* lipopolysaccharides (LPS) by use of a macroscopic tube agglutination test, a rapid serum plate agglutination (SPA) test, a stained antigen whole blood test, or a micro-agglutination test (Shivaprasad, 2000). Due to the fact that breeding flocks are screened for specific serum antibodies against *Salmonella* LPS using the SPA test (Gast, 1997), antibodies produced following vaccination are indistinguishable from those produced in response to a wild type *Salmonella* Pullorum infection. A central goal in ideal vaccine development is that it should not interfere with this salmonellosis monitoring program. The concept of DIVA vaccines based on the absence of at least one immunogenic protein or antigen in the vaccine, but which is present in the wild type strain, has already been proposed for commercial veterinary use (Selke et al., 2007; Leyman et al., 2011; Román et al., 2012). Moreover, the ability of live vaccine to shed and to persist in the environment should be tested to provide information for assessing the unacceptable hazard of the prolonged survival to the environment (Leyman et al., 2012).

The need to remove wild type genes in order to distinguish vaccinated strains from wild type strains offer new opportunities. In this regard, the construction of novel attenuated *Salmonella* vaccine strains has focused both on the deletion of virulence factors as well as the disruption of metabolic pathways, while at the same time balancing safety and immunogenicity (Galen and Curtiss, 2014). LPS is a major virulence factor and the *O*-antigen is the immunodominant antigen in serological diagnosis tests. Truncating the *O*-antigen and preventing its synthesis are two strategies for attenuating *Salmonella* for use as a DIVA vaccine (Kong et al., 2011). WaaL is a membrane enzyme implicated in ligating the *O*-antigen to the lipid A-core oligosaccharide. The presence or absence of the O-chain antigen determines whether the LPS is smooth or rough respectively. Mutations in the *waaL* gene result in bacteria possessing a rough LPS (Pérez et al., 2008). However, the *waaL* deletion mutant (such as *Salmonella* Typhimurium Δ*waaL*) has been found to be insufficiently attenuated (Nagy et al., 2008) and so a second virulence gene was thought to be required. SpiC is required for translocation of SPI-2 (*Salmonella* pathogenicity island 2) effectors (Geng et al., 2014) and so *spiC*, was selected as an additional attenuating deletion.

The objectives of the present study were to evaluate the safety and protective capacity of a *Salmonella* Pullorum Δ*spiC*Δ*waaL* double-mutant strain following intramuscular inoculation of day-old broilers and to evaluate the usefulness of this strain as DIVA strategy.

## Materials and Methods

### Chickens

HY-line white chicken embryos, obtained from Jiangsu Institute of Poultry Sciences (China), were hatched in the laboratory. The chickens were checked to confirm the absence of *Salmonella* infection by bacteriological examination as described below and for any clinical signs of enteric disease. Experimental groups were housed in separate wire cages with sufficient formulated feed and water at ambient temperature. All experimental and animal management procedures were permitted by the Animal Welfare and Ethics Committees of Yangzhou University (SYXK [Su] 2012–0029), and complied with the guidelines of the institutional administrative committee and ethics committee of laboratory animals. All laboratory health and safety procedures were complied with during the course of the experimental work.

### Bacterial Strains and Development of *Salmonella* Pullorum Mutant Strain

The *Salmonella* Pullorum S06004 strain, originally isolated from chicks with pullorum disease, is naturally resistant to nalidixic acid (Nal) (Geng et al., 2014), and was used as the challenge strain. A *spiC* deletion mutant strain, S06004Δ*spiC* (Guo et al., 2016), was used as the background strain for the construction of the LPS rough mutant. Deletion of the *waaL* gene was performed using the one-step inactivation method as described previously (Datsenko and Wanner, 2000). The targeted gene was deleted from start to stop codon, as confirmed by sequencing. The *Salmonella* Pullorum S06004Δ*spiC*Δ*waaL* mutant strain was used for immunization of chicks. The *Salmonella* Gallinarum virulent strain SG9 Nal^R^, supplied by Barrow (1990), and *Salmonella* Enteritidis F06 Nal^R^, isolated from dead poultry farm chickens, were used as challenge strains.

### LPS Extraction, Silver Staining and Immunoblot Analysis

Lipopolysaccharides was extracted by phenolic based extraction method using LPS extraction kit (iNtRON, Korea), the procedure involved lysis, precipitation, purification and resuspension. LPS samples were separated on 12% SDS-PAGE gels (sodium dodecyl sulfate polyacrylamide gel electrophoresis) and stained using a silver stain kit (Thermo Scientific, USA). Western blotting was performed using an anti-O9 monoclonal antibody and goat anti-mouse IgG HRP (1:5000; Sigma–Aldrich, USA). An acriflavine slide test was carried out to detect the rough colony phenotype characteristic of the mutant strain (Cerdà-Cuéllar and Aragon, 2008). Briefly, Colonies of a 24-h culture prepared from the vaccine strain on nutrient agar plates were mixed with 30 microlitres of 0.2% acriflavine on a glass slide. Mixtures were rotated for 1 min and observed for macroscopic evidence of agglutination.

### Assessment of LD_50_ and Organ Colonization

For determination of the 50% lethal dose (LD_50_), groups of 10-day-old chicks were infected intramuscularly with 10-fold dilutions (from 10^5^ to 10^10^ CFU) of S06004Δ*spiC*Δ*waaL* or S06004. Birds were observed for 3 weeks post-infection, and deaths were recorded daily to calculate the LD_50_ values.

Bacterial persistence and clearance were analyzed to determine the number of live vaccine strain by counting Nal resistant *Salmonella* per gram of liver or spleen. Forty chicks obtained on the day of hatching, were randomly divided into two equal groups. The vaccinated group was injected intramuscularly with 1 × 10^7^ CFU live vaccine, whereas the control group received 100 μl PBS. Following on from this, 2, 7, 14, and 21 days post vaccination, the liver and spleen were obtained from each group of five birds using aseptic technique, weighed, and then homogenized. Dilutions of the homogenate were incubated on selective XLT4 (Difco, USA) agar containing 100 μg/ml Nal at 37°C and the resulting *Salmonella* colonies were counted.

### Vaccination Experiments

#### Immunization and Protection against *Salmonella* Pullorum Challenge

Forty day-old chicks were divided into two groups (*n* = 20/group), designated as group A (non-vaccinated control) and group B (vaccinated), to evaluate the protective efficacy of the *Salmonella* Pullorum mutant following a single-dose intramuscular injection. These two groups of day-old chicks were injected with 100 μl of PBS (pH 7.2) or PBS containing ∼1 × 10^7^ CFU of the S06004Δ*spiC*Δ*waaL* mutant strain. Animals were challenged intramuscularly with 10^9^ CFU of virulent *Salmonella* Pullorum 14 days post-immunization (dpi) (Guo et al., 2016). Deaths and clinical symptoms were recorded daily for a further 2 weeks. At the end of this period, all surviving chicks were sacrificed. Clinical scores were determined and recorded using a system as we have previously described Guo et al. (2016). Organs (liver, spleen, and cecum) were collected from each group of five birds aseptically, weighed, and homogenized to check for the presence of the challenge strain. Dilutions of the homogenate were made in sterile PBS (pH 7.2) and plated onto selective XLT4 (Difco, USA) agar containing 100 μg/ml Nal.

#### Cross-Protection against Homologous Serotype

Four groups of 20 day-old chicks were vaccinated with S06004Δ*spiC*Δ*waaL* as described in Section “Immunization and Protection against *Salmonella* Pullorum Challenge.” At 50 days of age, the chicks were injected intramuscularly with 10^9^ CFU of the virulent *Salmonella* Gallinarum strain SG9 or with 10^9^ CFU *Salmonella* Enteritidis F06. Mortality was recorded over a period of 2 weeks.

Protection against *Salmonella* Enteritidis induced by the vaccine strain was assessed by analyzing the ability of the challenge strain to colonize the gastrointestinal tract, liver, and spleen of vaccinated and control chickens. Fourteen days after challenge, five birds from each group were sacrificed and tissue homogenates were prepared. Appropriate dilutions of the homogenates were plated on XLT4 agar plates containing 100 μg/ml Nal for the enumeration of *Salmonella* Enteritidis present in each tissue.

### Production of Antibodies and Lymphocyte Stimulation Assay

An indirect-ELISA method using heat-killed whole *Salmonella* Pullorum bacteria as coating antigen was applied to quantify IgG in the serum, as described previously (Guo et al., 2016). Briefly, bacterial antigen was coated in 96-well plate and incubated overnight at 4°C. After blocking, the sera samples collected on days 7, 14, 21, and 28 were diluted and added to the wells. Wells were incubated with a 1:5000 dilution of horseradish peroxidase-conjugated goat anti-chicken IgG secondary antibody (Sigma–Aldrich, USA) for 1 h. Absorbance at 405 nm was measured with an automated microplate reader (Synergy 2, BioTek, USA) after the reaction was stopped with 2 M H_2_SO_4_. The optical density (OD) values obtained were used to calculate the adjusted *E*-values using the following formula: *E*-value = [OD (sample) – OD (negative control)/OD (positive control) – OD (negative control)]. To evaluate the impact of the vaccine candidate on the cell-mediated immune (CMI) response post-immunization, a peripheral lymphocyte proliferation assay was carried out on day 21 as described previously (Rana and Kulshreshtha, 2006).

### Capability of DIVA Based Serodiagnosis

The SPA test was used to assess the DIVA capability of the S06004Δ*spiC*Δ*waaL* vaccine candidate (Gast, 1997; Lalsiamthara et al., 2015). The commercially available plate test *Salmonella* Pullorum antigens were obtained from Zhonghai Biotech (Beijing, China). Day-old chicks were immunized intramuscularly with the vaccine strain or the S06004Δ*spiC* mutant (smooth LPS phenotype). Serum were prepared on days 7, 14, 21, and 28 by collecting blood via heart puncture and allowing it to coagulate for 3 h at room temperature before collection of the serum fraction. Serum samples from vaccinated birds (30 μl) were mixed with an equal volume of standard SPA test antigen and the agglutination reaction was observed.

### Environmental Safety Evaluation

The sensitivity of the S06004Δ*spiC*Δ*waaL* mutant to ultraviolet light and various disinfection agents was evaluated as described (Leyman et al., 2012; Han et al., 2014), with minor modifications. Bacteria were grown to OD_600_ = 1.0 at 37°C with shaking and were diluted in PBS to 10^9^ CFU/ml. One milliliter of suspension was then transferred to a sterile petri dish and exposed to ultraviolet radiation (PHILIPS, UV-C: 254 nm, 30 W, distance: 45 cm) for 1 min. For oxidative and alkali tolerance, each suspension was incubated in the presence of stressors at room temperature: 100 μM hydrogen peroxide for 5 min or 12.5 mM sodium hydroxide (NaOH) for 5 min. The susceptibility of strains to compound peroxymonosulphate powder, which is the active ingredient in a selected disinfectant (DuPont^TM^ Virkon^®^ S, Shenzhen, China) was analyzed by a microdilution assay using serial, two-fold dilutions. Finally, 10-fold dilutions were plated onto LB plates to quantify viable bacteria. The experiment was performed three times.

### Statistical Analysis

Statistical analysis was performed using GraphPad Prism 5 (GraphPad Software, USA). One-way ANOVA followed by a Dunnett’s multiple comparison tests was used to determine the statistical differences between multiple experimental groups. *P* < 0.05 was considered statistically significant.

## Results

### The *waaL* Mutant Shows Truncated LPS Lacking the *O*-Antigen Region

**FIGURE 1:**
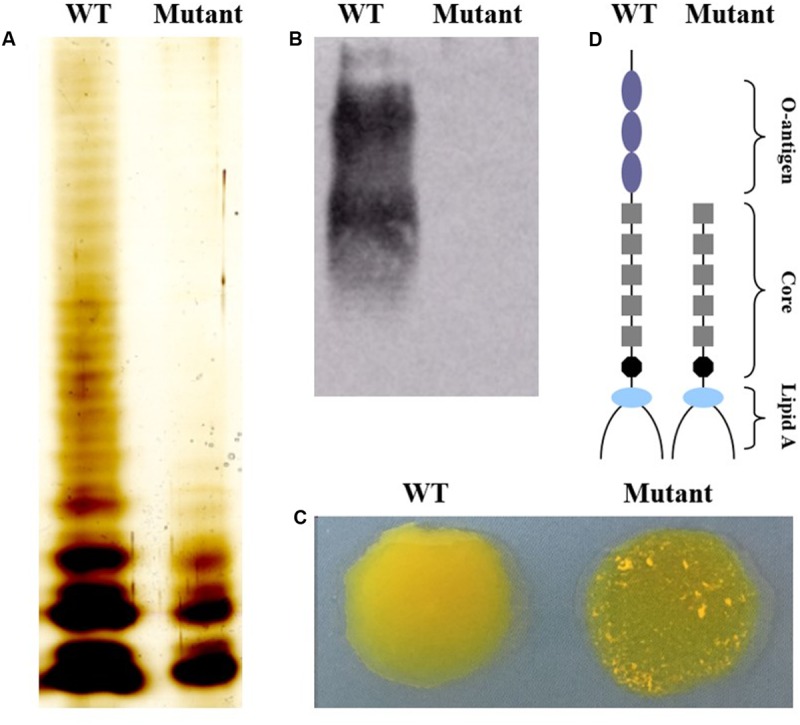
**Phenotypic characterization of LPS in mutant and wild type strains.** **(A)** SDS-PAGE of LPS analyzed by silver staining. **(B)** Western blotting analysis of LPS using an anti-O9 monoclonal antibody. **(C)** Mutant strain has a rough appearance using the acriflavine agglutination test. **(D)** Schematic representation of the *Salmonella* Pullorum LPS molecule (lipid A, core polysaccharides and *O*-antigen).

To confirm that *O*-antigen biosynthesis was affected, the LPS profiles of wild type and mutant strains were compared using silver staining of LPS preparations. As shown in **Figure 1A**, the LPS from the wild type strain displayed a ladder-like pattern with varying lengths of *O*-polysaccharide repeats, whereas the mutant with the *waaL* gene had a rough type A (Ra) LPS structure. Furthermore, western blotting confirmed the lack of reactivity of the *waaL* mutant LPS with an anti-O9 monoclonal antibody indicating that the LPS lacked the *O*-antigen (**Figure 1B**). The wild type strain and the *waaL* mutant strain could be distinguished from each other using the acriflavine slide test. The mutant strain displayed a rough-phenotype whereas the wild type strain displayed smooth-phenotype since it did not cause agglutination (**Figure 1C**).

### Determination of the Attenuation of Δ*spiC*Δ*waaL* Strain

In order to confirm the safety of the mutant strain as a live vaccine candidate, the virulence of parent and mutant strains were evaluated in day-old HY-line white chicks. The LD_50_ of the S06004Δ*spiC*Δ*waaL* was approximately 400-fold higher than that of the parent strain for intramuscular infection (**Table 1**).

**Table 1:** Characteristics of *Salmonella* Pullorum mutants.

Strain	Morphology	LD_50_ (CFU) ^a^
S06004	Smooth	1.0 × 10^7^
S06004Δ*spiC*	Smooth	1.20 × 10^9^
S06004Δ*spiC*Δ*waaL*	Rough	3.98 × 10^9^


*^a^Chicks were infected intramuscularly with the mutant strains.*

### Organ Colonization and Persistence

**FIGURE 2:**
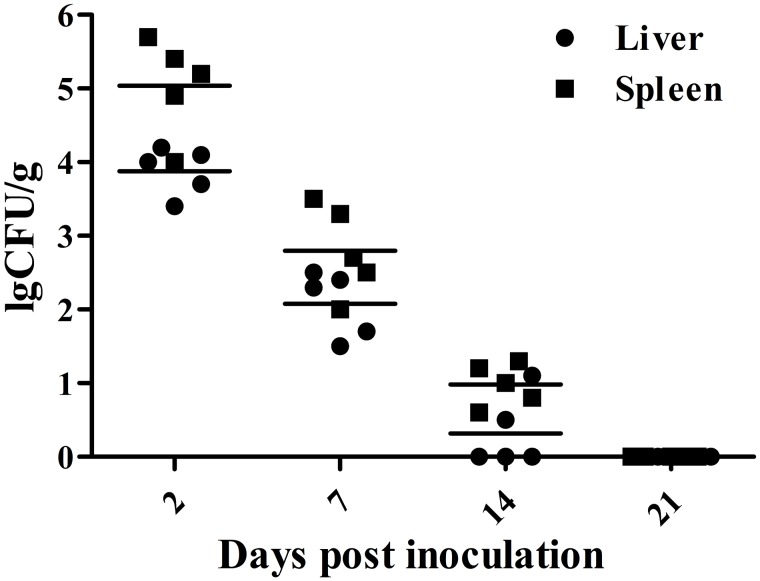
**Colonization and persistence of the vaccine strain in liver and spleen after inoculation of day-old chicks with 10^7^ CFU bacterial cells.** Values are represented as log_10_ CFU/g sample. Negative samples are shown as 0 CFU/g; all samples from the control group were negative.

The results of bacteria persistence and clearance in organs are shown in **Figure 2**. Bacteriological analysis showed that inoculation with the mutant strain resulted in a high level of internal organ colonization 2 days post-vaccination and persistence for about 2 weeks, suggesting the vaccine strain maintained the ability to invade the host and to stimulate an immune response. In general, however, the persistence of the strain declined gradually over the course of the experiment. All samples taken 21 days after inoculation were negative, indicating that the mutant strain was efficiently cleared.

### Protective Efficacy against Virulent *Salmonella* Pullorum Challenge

**FIGURE 3:**
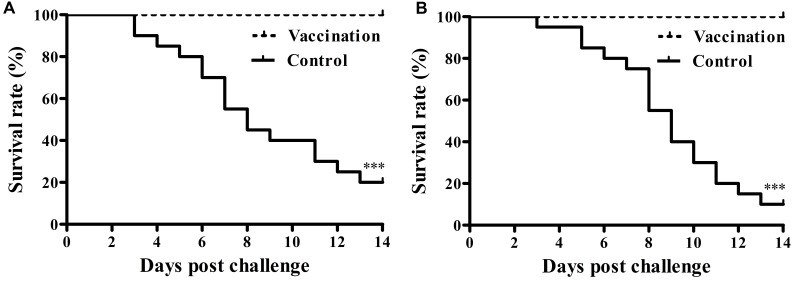
**Protective ability of the rough mutant against the virulent *Salmonella enterica* serovar Gallinarum biovar Pullorum and Gallinarum challenge.** At fourteen dpi, chicks from each group were challenged intramuscularly with 1 × 10^9^ CFU wild-type *Salmonella* Pullorum S06004 **(A)** or virulent *Salmonella* Gallinarum SG9 **(B)**. Protection was expressed by percentage survival after challenge (^∗∗∗^*P* < 0.001).

Chicks that were inoculated with the vaccine showed significantly higher protection against *Salmonella* Pullorum challenge compared to non-immunized chicks. No mortality was observed in the immunized group whereas 16 out of 20 broilers died in the non-vaccinated group (**Figure 3A**). Post-challenge, the mean pathology scores of the vaccinated group and the control group were 1.10 ± 1.77 and 4.70 ± 0.66, respectively, indicating that immunized chicks received significantly lower mean scores than controls. The vaccinated birds also had a significant reduction in challenge bacterial counts in the organs (**Table 2**).

**Table 2:** Recovery of *Salmonella* from various chick organs vaccinated with the mutant strain or non-immunized control chicks subsequently challenged with virulent *Salmonella*.

Treatment	*Salmonella* Pullorum (lgCFU/g)			*Salmonella* Enteritidis (lgCFU/g)		
		
	Liver	Spleen	Cecum	Liver	Spleen	Cecum
Vaccination	0.92 ± 0.15^a^	2.11 ± 0.53^a^	4.04 ± 0.23^a^	–^a,b^	–^a,b^	–^a,b^
Control	3.40 ± 0.49	4.09 ± 0.14	5.57 ± 0.41	1.16 ± 0.36	3.0 ± 0.34	4.23 ± 0.65


*^a^*P* < 0.01 compared to PBS controls. ^b^No *Salmonella* Enteritidis challenge strain detected.*

### Cross-Protection against Virulent *Salmonella* Gallinarum or *Salmonella* Enteritidis Challenge

To evaluate the cross-protective effect of the live vaccine, vaccinated chicks were challenged with the virulent *Salmonella* Gallinarum strain SG9 or the *Salmonella* Enteritidis strain F06. Following challenge with virulent SG9, none of the chicks died in the vaccinated group, whereas only two chicks survived in the non-vaccinated group (**Figure 3B**). Bacteriological analysis of the tissues from vaccinated birds following virulent *Salmonella* Enteritidis challenge showed that the wild type strain could not be detected in either systemic organs or the cecum; this was not the case in the control group (**Table 2**).

### Humoral and Cellular Immune Response Generated by the Vaccine Candidate

**FIGURE 4:**
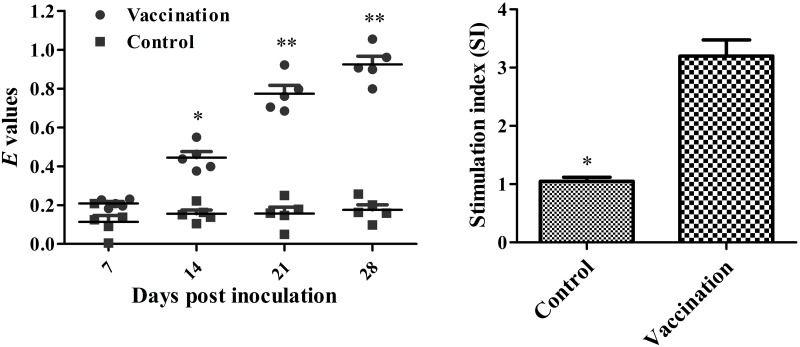
**Levels of humoral and cellular immune responses in vaccinated versus control chicks.** Serum IgG antibody responses against the live vaccine strain measured by indirect-ELISA. Asterisks indicate a significant difference between the immunized and PBS control groups (^∗^*P* < 0.05; ^∗∗^*P* < 0.01). Stimulation index of lymphocyte stimulation responses using soluble antigens in immunized chickens at 3 weeks post vaccination. Asterisks indicate a significant difference compared with the PBS control group (^∗^*P* < 0.05).

Statistical analysis showed a significant difference in both the humoral and cellular immune response in chickens immunized with the vaccine compared with the control animals. Serum IgG levels were significantly higher at every time point post-immunization in the treatment group (**Figure 4**). In vaccinated chicks, the level of the lymphocyte proliferative response, indicated by the stimulation index (SI), was significantly higher compared to control chicks (**Figure 4**).

### Differentiation of Serum Antibodies to *Salmonella* Pullorum

**FIGURE 5:**
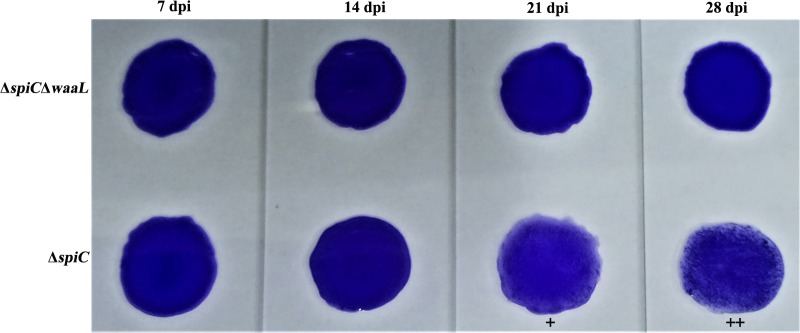
**Capability of DIVA based on a serum plate agglutination test.** Serum collected on days 7, 14, 21, and 28 were tested for reactivity to the *Salmonella* Pullorum standard antigen. Chicks in the S06004Δ*spiC* control group showed a strong agglutination reaction (+/++) on days 21 and 28 post inoculation.

Antibodies to *Salmonella* Pullorum are often detected by the SPA test using the standard antigen. Serum collected from S06004Δ*spiC*Δ*waaL* vaccinated chicks at different time intervals was identified as SPA negative post-inoculation (**Figure 5**). However, chicks were identified as positive in the group vaccinated with the *spiC* strain. Overall, the live vaccine has shown obvious DIVA capability which could be extremely useful in the salmonellosis control program.

### The Mutant Strain Shows Enhanced Sensitivity to Environmental Stress

**FIGURE 6:**
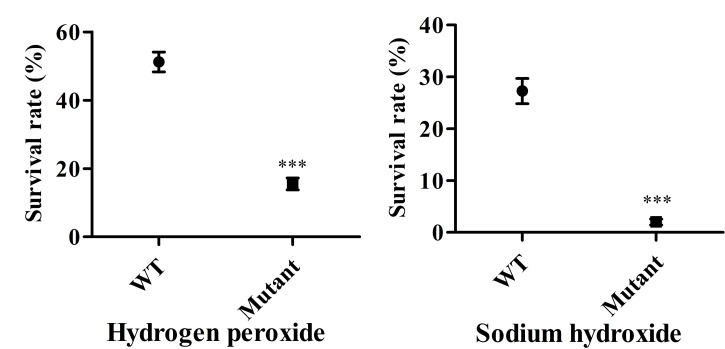
**Mutant strain survival rates under different environmental stress.** The susceptibility of the *Salmonella* Pullorum mutant strain and its parental strain to hydrogen peroxide (100 μM for 5 min) and 12.5 mM NaOH for 5 min. Data are the means and standard deviations of three experiments (^∗∗∗^*P* < 0.001).

High UV sensitivity was demonstrated in both wild type and attenuated vaccine strains with no cells from either surviving after UV-C irradiation. In terms of oxidative stress, the percent survival of the mutant strain fell by approximately 84.5% and they demonstrated a markedly higher sensitivity to hydrogen peroxide than wild type cells (48.8%) (**Figure 6**). The LPS deficient strain was highly susceptible to alkaline treatment compared to the corresponding wild type strain (**Figure 6**). The minimum bactericidal concentration analysis showed that the rough mutant was much more sensitive to the killing effects of compound peroxymonosulphate powder than the wild type strain, with a significant reduction in number of surviving cells at some range of concentrations (**Table 3**). Consequently, the live vaccine candidate was unable to persist under adverse conditions, suggesting that it could be easily eliminated from the environment.

**Table 3:** Susceptibility of *Salmonella* Pullorum mutant strain to peroxymonosulphate powder.

Strain	Cell counts			
	
	1:200	1:400	1:800	1:1600
WT	0	182 ± 88.50	–^a^	–^a^
Mutant	0	0	16 ± 11.79	–^a^


*^a^Not counted.*

## Discussion

*Salmonella* Pullorum causes outbreaks in poultry, resulting in high chick morbidity and mortality (Xiong et al., 2016). Vaccination is not a panacea but should always be used as a part of a comprehensive control program. Live attenuated vaccines have been tested extensively in poultry and also in mice, although only a few *Salmonella* strains are registered and commercially available for use in poultry (Barrow, 2007). Ideally, a vaccine used to control *Salmonella* infection should be safe, cross-protective, well defined, and capable of inducing cellular immunity (Desin et al., 2013).

Genetic modification of the *Salmonella* vaccine strain aims at reducing the risk of persistence or spread in host tissues and in the environment while at the same time inducing an efficient immune response. Double mutations introduced into bacteria can develop a highly attenuated and safety-enhanced vaccine strain and thereby reduce the risk of reversion by acquisition of genes through horizontal transfer (Van Immerseel et al., 2005). The *O*-antigen provides important elements in serological distinction and plays a direct role in virulence such as invasion, colonization, and resistance. The WaaL protein is known to be involved in the ligation of *O*-antigen to lipid A-core oligosaccharide (Kaniuk et al., 2004). The *waaL* mutant in our vaccine strain produces rough LPS lacking the *O*-antigen repeat units (**Figure 1**). SPI-2 T3SS (type III secretion system) is involved in the avoidance of or resistance to macrophage killing mechanisms, SpiC is part of a three-protein regulatory complex that functions within *Salmonella* to mediate the switch from translocon protein secretion to effector translocation (Figueira and Holden, 2012). Because of this central role, single deletion of the *spiC* gene results in significant attenuation in *Salmonella* Pullorum. Our results demonstrate that the S06004Δ*spiC*Δ*waaL* double mutant was highly avirulent in day-old chicks because it was unable to cause mortality even at the high bacterial loads used for vaccination.

Bacterial susceptibility to different disinfection agents is an important measure of the fitness of a vaccine strain to survive in the environment (Leyman et al., 2012). LPS plays an important role in bacterial defense mechanisms against antimicrobial substances (Liu et al., 2014). *O*-antigen is the characteristic polysaccharide of the outermost component of the outer membrane of the gram-negative bacteria cell wall. The presence of *O*-polysaccharide in LPS molecules may prevent antimicrobial substances from reaching their binding sites in the cell wall, resulting in enhanced tolerance and survival. The mutant strain we have developed is highly susceptible to various environmental stresses, indicating that the live vaccine candidate will be likely be unable to persist under adverse conditions and will be easily eliminated from the environment. In addition to this, the vaccine strain was eliminated from the broilers before slaughter age, therefore showing a good safety profile. These results indicate that the vaccine candidate is sufficiently safe for chicks.

Besides safety, the efficacy of the vaccine is also important. Humoral and cellular immune responses induced by *Salmonella* vaccine play an important role in preventing infection (Penha Filho et al., 2012). Measurement of the levels of *Salmonella* specific antibodies is used to monitor humoral immune responses. Chicks from the immunized group had significantly higher serum IgG levels compared with chicks in the control group. The role of CMI in *Salmonella* resistance has been shown to involve both the acquired and innate immune responses. Furthermore, enhanced CMI may prove beneficial in protecting avian species against these intracellular bacterial infections (Babu et al., 2003). Measurement of CMI in vaccinated birds was carried out to investigate the effects of the vaccine on peripheral lymphocyte proliferation. In this study, the live vaccine was effective in increasing peripheral lymphocyte proliferation in response to the *Salmonella* Pullorum soluble antigen. Overall, the vaccine strain induced a robust humoral immune response as well as an effective CMI response.

*Salmonella* Pullorum, *Salmonella* Gallinarum and *Salmonella* Enteritidis all belong to the *Salmonella* serogroup D and cross-protection of the vaccine is therefore to be expected. Bacterial mutants with a truncated LPS, like the mutant used here, may have the potential to serve as a vaccine that generates cross-protective immunity to other serovars (Nagy et al., 2008; Kong et al., 2011; Leyman et al., 2011). The rough strain of *Salmonella* Gallinarum 9R has been used as a vaccine to protect chickens against fowl typhoid. Besides controlling fowl typhoid, the SG 9R vaccine has been used in several countries as an additional tool in the control of *Salmonella* Enteritidis in commercial layer flocks (Wigley, 2017). Recently, it has been suggested that rough mutants provide better accessibility of lipid A and core antigens, while at the same time they facilitate refocusing of the immune response to outer membrane proteins (Bearson et al., 2016; Liu et al., 2016). Therefore, a cross-protection study was carried out to investigate the ability of the rough *Salmonella* Pullorum vaccine candidate on cross-immunity. The mutant strain was able to provide cross-protection following challenges with wild type *Salmonella* Gallinarum by reducing mortality and with wild type *Salmonella* Enteritidis by preventing colonization. As more than one serogroups of *Salmonella* in the poultry community, we will evaluate the cross-protective immunity and protection efficacy against heterologous serotype *Salmonella*, such as *Salmonella* Typhimurium and *Salmonella* Infantis, in future works.

The basis for successful control of *Salmonella* Pullorum infections in poultry are good farming and hygienic practices as well as testing and removal of positive flocks from populations. When vaccination is used in *Salmonella* control programs, possible interferences with the standard *Salmonella* serological detection methods are considered to be a major drawback since the immunized animals produce antibodies against the vaccine strain that cannot be distinguished by serological tests from animals infected with wild type strains (Adriaensen et al., 2007). The success of an LPS based DIVA vaccine (*Salmonella* Gallinarum 9R) in the control of fowl typhoid and paratyphoid has shown that live attenuated, but distinguishable, bacterial strains provide evidence of a path forward (Silva et al., 1981). *Salmonella* Gallinarum 9R is a mutation in the *rfaJ* gene and results in a rough LPS phenotype because of a defective *O*-antigen side chain (Kwon and Cho, 2011). One of the greatest advantages of the use of the 9R vaccine is that it gives good protection and does not interfere with the tests used for pullorum-typhoid control. In the present study, the *waaL* gene mutant displayed a truncated LPS lacking the main surface antigen, similar to *Salmonella* Gallinarum 9R. The serologic results obtained from vaccinated birds were negative, however, the samples tested positive in wild type infected animals. Our results illustrate that the S06004Δ*spiC*Δ*waaL* mutant strain was able to stimulate a DIVA humoral immune response in chickens and show a satisfactory performance in the serologic tests in the *Salmonella* control program.

In summary, we have demonstrated that the rough attenuated *Salmonella* Pullorum vaccine candidate had attributes desirable in a vaccine including, attenuation, immunogenicity, and DIVA feature. This mutant strain may therefore be an efficacious vaccine in controlling *Salmonella* infection in poultry. Further efforts are necessary to determine the vaccine efficacy in field trials and to develop efficient control strategy against salmonellosis.

## Author Contributions

XJ, SG, and RG conceived and designed the study. ZP, XC, and QL conducted experiments. XF carried out sample collection. RG, YJ, and SZ carried out animal experiments. RG and YJ performed statistical analysis. RG and ZL drafted the manuscript. XJ finalized the manuscript. All authors read and approved the final version of the manuscript.

## Conflict of Interest Statement

The authors declare that the research was conducted in the absence of any commercial or financial relationships that could be construed as a potential conflict of interest.
